# Queue Gaps Among the IQGAPs in *Dictyostelium discoideum*

**DOI:** 10.3390/ijms27125462

**Published:** 2026-06-17

**Authors:** Vedrana Filić, Igor Weber

**Affiliations:** Division of Molecular Biology, Ruđer Bošković Institute, Bijenička 54, 10000 Zagreb, Croatia; vedrana.filic@irb.hr

**Keywords:** IQGAP, scaffold proteins, Rho GTPases, GAP1 protein family, actin cytoskeleton, cell migration, cell adhesion, macroendocytosis, cell division, *Dictyostelium*

## Abstract

Based on their domain organisation, four proteins from the protist *Dictyostelium discoideum* have been assigned to the IQGAP family of scaffold proteins. Although these proteins are shorter than animal IQGAPs, their involvement in the regulation of the actin cytoskeleton in cell motility, macroendocytosis, cytokinesis, and adhesion appears to be broadly conserved between these evolutionarily distant organisms. In this article, we show that the putative three-dimensional structure of *Dictyostelium* IQGAP-related proteins, as predicted by AlphaFold 3, closely corresponds to the C-terminal half of human IQGAP1, thus supporting their common origin. IqgD is the largest IQGAP-related protein in *Dictyostelium*, with an overall domain organisation similar to human IQGAPs. IqgD is localised in the cell cortex, interacts with F-actin and Rac1 GTPases, and primarily supports cell adhesion to the underlying surface and cell growth on bacterial lawns. DGAP1 and GAPA are truncated proteins that have retained a 700-residue-long C-terminal region of homology compared to their animal relatives. They play important, yet opposite, roles in regulating contractile cortical assemblies comprising F-actin, myosin II, and the actin-bundling proteins cortexillins, which are especially important for cytokinesis and epithelial morphogenesis. Finally, IqgC, although structurally resembling other IQGAPs, turns out to be more closely related to GAP1 proteins from fungi. This multifaceted protein carries RasGAP activity, interacts with several other small GTPases, and positively regulates macroendocytosis and cell–substratum adhesion.

## 1. IQGAP Family of Multidomain Scaffold Proteins

Proteins from the IQGAP family have emerged as typical pleiotropic scaffold proteins [1,2,3]. Scaffold proteins primarily serve as platforms that facilitate the co-localisation and interaction of multiple proteins, thereby contributing to the spatial organisation of the cellular interactome [4,5]. IQGAPs are multidomain proteins with seven well-established protein-binding domains, which interact with specific domains of other proteins but lack enzymatic activity [1]. From the N-terminus, these domains are calponin homology domain (CHD), coiled coil repeat region (CC), tryptophan-containing proline-rich motif-binding region (WW), an IQ domain containing several isoleucine- and glutamine-rich (IQ) motifs, GTPase-activating protein (GAP)-related domain (GRD), RasGAP C-terminal (RGCt) domain, and extreme C-terminal (CT) domain. The interactomes of human IQGAPs have been comprehensively described in recent review articles [6,7].

There is considerable inconsistency in the literature regarding the names and boundaries of the domains in the C-terminal halves of animal IQGAP proteins. Researchers typically define domains based on similarities in their linear sequences—both within the protein group and with related proteins, such as RasGAPs—or on the sizes of constructs used in interaction studies. Most papers consider amino acid residues 1025–1237 to constitute the primitive GRD [3,6,8,9], while the Ahmadian group defines the GRD as contiguous with RGCt, covering residues 962–1344 [10]. There is even greater variability regarding the RGCt domain. Located downstream of the GRD, the RGCt domain is generally regarded as the C-terminal protein interaction region of IQGAPs that mediates binding to Rho family GTPases, phosphoinositides, and cytoskeletal and adhesion proteins [1,11,12]. Some of the best-characterised RGCt binding partners, including E-cadherin [13], β-catenin [13], CLIP-170 [14], APC [15], Dia1 [16], PIP_2_ [17], Rac1, and Cdc42 [10], interact with motifs within the 150 amino acid residues at the C-terminus, which was initially designated as the RGCt domain [11]. However, in the following years, RGCt was inconsistently defined, mostly as amino acid residues 1451–1583 [3,6,8,9], or as almost everything downstream of the GRD (1276–1657) [1]. Recently, the Ahmadian group defined RGCt as amino acid residues 1345–1563 and introduced the concept of a separate CT domain at the extreme C-terminus (1576–1657) [10,12].

## 2. Re-Evaluation of the IQGAP Domain Organisation by AlphaFold 3

### 2.1. AlphaFold 3 Predicts the Tertiary Structure of the C-Terminal Half of IQGAP1 with High Confidence

**Figure 1:**
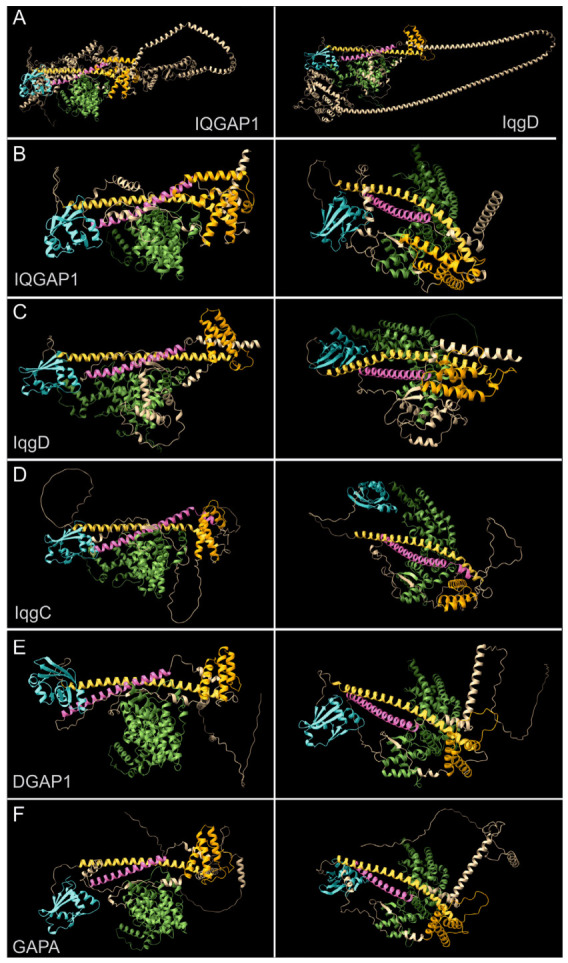
Computationally predicted structures of human IQGAP1 and IQGAP-related proteins from *Dictyostelium discoideum*. (**A**) Predicted three-dimensional structures of full-length IQGAP1 (left) and IqgD (right) displayed at a reduced scale; (**B**) C-terminal part of IQGAP1 (amino acid residues 836–1657); (**C**) C-terminal part of IqgD (amino acid residues 561–1385); (**D**) full-length IqgC; (**E**) full-length DGAP1; (**F**) full-length GAPA. In (**B**–**F**), two projections are shown side by side in the left and right columns. Individual structural features are coloured as follows: N-terminal helix (HN) in pink, GAP-related domain (GRD) in green, triplet of short helices within the RasGAP C-terminal (RGCt) domain (RGCt-H3) in orange, C-terminal helix within the RGCt domain (RGCt-HC) in yellow, and the C-terminal domain (CT) in cyan. The remainder of the protein is coloured beige. The intervals of amino acid residues corresponding to each domain are listed in Table 1. Predicted three-dimensional structures were obtained using the AlphaFold web server powered by the AlphaFold 3 model [21]. Visualisation was performed with UCSF ChimeraX [22]. The primary protein sequences were downloaded from the UniProt website [23].

Structural data provide the most accurate basis for defining protein domains. However, experimentally determined three-dimensional structures are currently available only for the GRD and CT domains—the latter also termed atypical phosphoinositide binding (aPI)—of IQGAP1 and IQGAP2, respectively [18,19]. Therefore, we used computational predictions of the 3D structures of IQGAP1 and related proteins from *Dictyostelium* as a basis for working definitions of domains in their C-terminal regions. The results obtained using the biomolecular structure prediction tool AlphaFold 3 are shown in Figure 1. The predicted structural domains, whose boundaries we define as the margins of prominent three-dimensional features, such as single extended α-helices or compact clusters of α-helices and β-sheets, are shown in colour in Figure 1 and listed in Table 1. AlphaFold utilises machine learning to predict a protein’s 3D structure based primarily on its primary amino acid sequence, using experimentally determined protein structures as training data. To evaluate the expected accuracy of a model’s local atomic distances compared to a true experimental structure, the predicted Local Distance Difference Test (pLDDT) confidence metric, scaled from 0 to 100, is used for each residue. Scores above 90 indicate very high confidence in a model’s prediction, including the amino acid side-chain orientations, whereas scores between 70 and 90 indicate that the backbone geometry of the model is highly reliable. pLDDT values below 50 typically designate intrinsically disordered or highly flexible regions [20]. The average pLDDT values for the predicted domains listed in Table 1 are shown in Table S1.

The predicted 3D structures and domain architectures are remarkably consistent among the five analysed proteins, despite significant differences in their linear amino acid sequences (21–30% identity between human IQGAP1 and *Dictyostelium* IQGAPs). As shown for IQGAP1 (Figure 1B), the GRD defined in this way corresponds to a large, elongated, crescent-shaped, all-helical cluster containing thirteen α-helices, whereas the CT corresponds to a smaller, compact cluster of four short α-helices and seven β-sheets. The pLDDT scores for these two domains are between 80 and 90 for all five analysed proteins, although experimentally determined structures of a GRD and a CT domain are available only for mammalian IQGAPs [18,19]. This indicates structural conservation of these two domains between mammalian and *Dictyostelium* proteins. The only exception is the GRD from IqgD, which has a 78-residue-long insertion between Asn1040 and Arg1117 that lowers its pLDDT score.

### 2.2. A Novel Prediction of the RGCt Three-Dimensional Structure

In contrast to the GRD and CT domains, no experimental data are available for the structure of the RGCt domain of IQGAPs. Based on the predicted structure of IQGAP1 obtained by AlphaFold 3, we hypothesise that this domain consists of two distinct structural features: a triplet of interconnected α-helices and a single extended α-helix, which we designate as RGCt-H3 and RGCt-HC, respectively. The pLDDT scores for these two subdomains are close to 80, indicating high confidence in their predicted backbone geometry; that is, the tertiary structure of the α-helical segments. The two RGCt subdomains are connected by a short linker of indeterminate structure (50 < pLDDT < 70). The predicted structure also indicates the presence of another elongated α-helix corresponding to a segment positioned upstream from the GRD in the primary sequence and juxtaposed to RGCt-HC in the predicted 3D structure, which we designate as HN (Figure 1). Although the average pLDDT values across the hypothetical HN are significantly lower than for RGCt-HC (Table S1), two additional computational results support this prediction. Predicted Aligned Error (PAE) is a metric that estimates the expected positional error in ångströms in the predicted distance between specific parts of an AlphaFold structure. The average PAE values obtained when the entire lengths of RGCt-HC and HN, as defined in Table 1, are considered are close to 7 Å. However, when only about 70% of both helices, covering the regions of their close juxtaposition, is considered, the PAE values drop below 5 Å, indicating high confidence in the relative distance and orientation between the two core segments of RGCt-HC and HN. In addition, these two helices from distant regions of the primary sequence probably form a coiled coil structure, as suggested by the high probability of heptad repeats occurring in both helices, predicted by the PCOILS algorithm (Figure S1) [24]. As far as we are aware, this is the first time this potential structural feature of IQGAP1 and related proteins has been described, and we suggest that it could play an important role in regulating their activity. According to the predicted structure, the well-structured domains are connected by hinges or loops of varying length and uncertain structure. Based on their low average pLDDT values, the linker regions between HN and GRD and between RGCt-HC and CT are predicted to represent Intrinsically Disordered Regions (IDRs; Table S1).

## 3. IQGAP-Related Proteins in *D. discoideum*

### 3.1. Evolutionary Perspective on the IQGAP-Related Proteins in D. discoideum

IQGAP representatives have been identified in animals, fungi, and protists [7,9] and share the characteristic domain composition of their C-terminus. Functional studies have shown that IQGAPs are primarily involved in regulating the actin cytoskeleton, but they also regulate other cellular functions [7,25]. Although the three IQGAP isoforms in humans share a similar domain architecture, their expression profiles are highly tissue-specific, and their functions are somewhat diversified [6,7]. IQGAPs represent one of the five clusters in the RasGAP domain-containing protein superfamily, likely originating from an ancestral gene in the Last Eukaryotic Common Ancestor (LECA) [26]. Moreover, IQGAPs and the GAP1 family of RasGAPs from fungi share a common origin predating the LECA. IQGAPs are unique within the RasGAP superfamily because their RasGAP domain has lost its activity due to mutations of critical residues and has thus been renamed the GAP-related domain (GRD) [18,27]. IQGAP-related proteins have also been identified in protists, which diverged from the animal lineage more than a billion years ago. The first protist IQGAPs were identified in *D. discoideum*. *D. discoideum* amoebae are highly motile cells with a complex actin cytoskeleton, and their lifestyle resembles that of mammalian leukocytes [28,29]. It is, therefore, of general interest to study how the highly divergent proteome of *D. discoideum* supports cellular traits similar to those of mammalian cells, which appears to be a prominent example of convergent evolution. In this review, we provide an overview of four IQGAP-related proteins in *D. discoideum* and compare their structure and function with those of mammalian IQGAPs.

The first two IQGAP-related proteins from protists were identified in *D. discoideum* soon after the discovery of the first human IQGAP in 1994 [30,31,32]. The *Dictyostelium* genome sequencing project identified two additional candidate IQGAP genes [33,34]. Subsequent efforts to streamline the original nomenclature resulted in multiple names for the four proteins: DGAP1 (DdIQGAP1, IQGAP1, rasGAP1, RIP2), GAPA (DdIQGAP2, IQGAP2, rasGAP), IqgC (DdIQGAP3), and IqgD (DdIQGAP4). In this article, we will adhere to the original designations. Whereas mammalian IQGAPs contain seven established domain types, there are five domain types in yeast and only three in *Dictyostelium* proteins, whereas IqgD has a CHD in addition. Whether these differences arose through the addition of new domains or the loss of those originally present in the common ancestor is currently unclear [26,35].

### 3.2. Tertiary Structure and Regulatory Motifs Are Conserved in IQGAP-Related Proteins in D. discoideum

**Figure 2:**
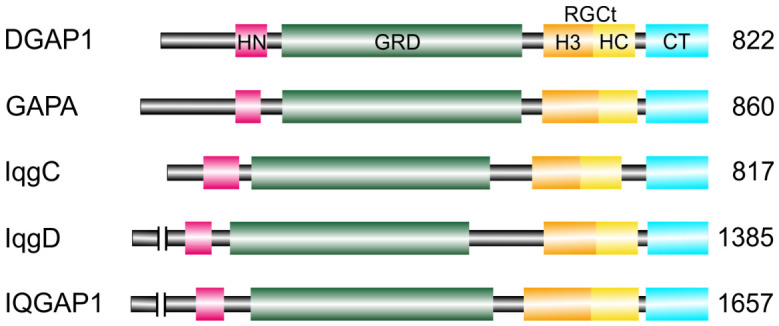
Schematic domain organisation of DGAP1, GAPA, IqgC, and the C-terminal halves of IqgD and human IQGAP1. The domain boundaries correspond to the margins of prominent features in the predicted three-dimensional protein structures. The colour code is the same as in Figure 1. The number of residues for each full-length protein is shown on the right-hand side.

The sizes and sequence-based domain organisation of DGAP1, GAPA, IqgC, and the corresponding C-terminal part of IqgD are roughly comparable to the C-terminal half of human IQGAP1. Consistently, their hypothetical tertiary structures, as predicted by AlphaFold 3, are remarkably similar (Figure 1). As in the predicted 3D architecture of IQGAP1 described above, predicted structures of four *Dictyostelium* proteins feature an HN helix followed by a crescent-shaped GRD, an RGCt consisting of an H3 triad and an HC helix, and a CT domain. Analysis by PCOILS also reveals a high propensity for HN and RGCt-HC to form a coiled coil superhelix in all four *Dictyostelium* proteins, as in human IQGAP1 and IQGAP2 (Figure S1). An additional cluster of three α-helices and two β-sheets is found between GRD and RGCt in IqgD (Figure 1C), and an α-helix is present at the N-termini of DGAP1 and GAPA (Figure 1E,F). In all predicted structures, individual domains and motifs are connected by segments of varying lengths with no apparent secondary structure and two hypothetical IDRs, as in IQGAP1 (Figure 2; Table S1).

In addition to spatial domain organisation, two regulatory mechanisms characteristic of mammalian IQGAPs appear to be conserved in *Dictyostelium* proteins. The first involves a polybasic motif responsible for the interaction of IQGAP1 with PIP_2_ [17]. In IQGAP1, this motif encompasses a cluster of six lysine residues between Lys1546 and Lys1558, located on an exposed loop, IDR2, between the RGCt and CT domains. Such polybasic clusters are present in all *Dictyostelium* proteins, with the number of lysine residues ranging from three in IqgD to thirteen in IqgC. The second putative regulatory mechanism is based on the phosphorylation of serine residues within the RGCt-H3 subdomain in IQGAP1. It has been shown that phosphorylation of IQGAP1 at S1443 strongly modulates its binding to Cdc42 [36]. In *Dictyostelium* proteins, serine residues are also located within the RGCt-H3 on the exposed hinge regions between individual α-helices. Although the conservation of the polybasic motif and exposed serines in *Dictyostelium* proteins suggests that the corresponding regulatory mechanisms are also conserved in this organism, appropriate experimental tests of this hypothesis have not yet been performed. Taken together, the predicted three-dimensional structures obtained by AlphaFold 3 suggest that the IQGAP-related proteins from *D. discoideum* share overall tertiary structure and common regulatory motifs with mammalian IQGAPs. Next, we turn to the specific functions of these proteins in the biology of *D. discoideum*.

## 4. IQGAPs Cut in Half: DGAP1 and GAPA

### 4.1. DGAP1 and GAPA Have Opposite Effects on the F-Actin/G-Actin Ratio in Cells

DGAP1 and GAPA were identified as IQGAP-related proteins because, like animal IQGAPs, they lack GAP activity towards Ras GTPases. They are also approximately half the length of IQGAP1, missing the entire N-terminal half of the animal proteins. Although they lack the actin-binding CHD found in animal and fungal IQGAPs, DGAP1 and GAPA are, nevertheless, implicated almost exclusively in the regulation of the actin cytoskeleton. The sequences of DGAP1 and GAPA are 51% identical and 67% similar to each other, and they share most interacting partners, but they play antagonistic roles in cell physiology. Upon binding Rac1 GTPases in their active, GTP-loaded form, both DGAP1 and GAPA interact with the actin-binding proteins cortexillins [37,38]. Other interaction partners of GAPA include filamin [38], myosin II [39], and α-catenin, which also interact with DGAP1 [40].

Cell motility and cell growth on bacterial lawns are inversely correlated with the expression level of DGAP1 [30,41,42]. Consistently, the ratio between F- and G-actin is increased in *dgap1^−^* cells, leading to increased production of actin protrusions, whereas it is decreased in DGAP1 overexpressor [41]. It was, therefore, hypothesised that DGAP1 sequesters active Rac1 and thereby reduces Rac1-stimulated actin polymerisation via WASP/WAVE-Arp2/3 complexes and formins [43,44]. Indeed, it was shown that single *dgap1^−^* and *gapA^−^* mutants contain higher levels of active Rac1 compared to wild-type cells and that this effect is roughly additive in the double null mutant [45]. Surprisingly, however, the ratio between F- and G-actin is decreased in *gapA^−^* cells, whereas it is increased in GAPA overexpressor [38]. It appears, therefore, that actin polymerisation is promoted by GAPA-Rac1 complexes, whereas it is suppressed by DGAP1-Rac1 complexes.

### 4.2. GAPA Promotes Mechanoresponsive Accumulation of Myosin II and Cortexillin I

In wild-type cells, both DGAP1 and GAPA form quaternary complexes with the small GTPase Rac1 and a dimer of actin-bundling proteins, cortexillins, which are necessary for efficient cytokinesis in *Dictyostelium* [37]. Given the high sequence similarity between the two proteins, it was puzzling that *gapA^−^* cells displayed a strong cytokinesis defect, whereas *dgap1^−^* cells did not [31,41]. However, cytokinesis was disrupted in DGAP1-overexpressing cells, while the *dgap1^−^*/*gapA^−^* cells exhibited a severe cytokinesis defect resembling that of cortexillin I/II double-null (*ctxA^−^*/*ctxB^−^*) cells [37,41]. The differences between the apparent roles of DGAP1 and GAPA in cytokinesis have been interpreted within the model of mechanoresponsive contractile protein complexes that determine the mechanical properties of the cell cortex [39]. According to this conceptual framework, the main components of these “contractility kits” are the mechanoenzyme myosin II as the force-generating component, the actin-bundling protein cortexillin I as the force-bearing component, and GAPA as a positive regulator [46]. Multiple lines of evidence suggest that DGAP1 inhibits the mechanoresponsive accumulation of myosin II and cortexillin I by suppressing the incorporation of GAPA into contractility kits [47,48]. This model explains why cells lacking the positive regulator of contractility, GAPA, have a strong cytokinesis defect, whereas those lacking its suppressor, DGAP1, do not.

During the culmination stage of the *D. discoideum* developmental cycle, differentiated cells at the tip of the multicellular fruiting body form a polarised epithelium that surrounds the stalk tube [49]. As in metazoan tubular epithelia, the apical side of the stalk tube cells is constricted by actomyosin activity relative to the basal side, forming wedge-shaped cells that enclose the tube. Interestingly, DGAP1 plays a major role in the apical localisation of myosin II. α-catenin, which is localised on basolateral membranes, recruits the DGAP1/cortexillin complex, which prevents myosin II from binding at those sites, resulting in selective localisation of myosin II to the apical cortex. Knockout of DGAP1 or cortexillin I results in mislocalisation of myosin II to the basolateral cortex, leading to disturbed morphology of the tip epithelium [50]. Thus, there is a clear analogy between the prohibitive role of DGAP1 in myosin II recruitment to specific domains of the cellular cortex during cytokinesis in the unicellular stage and during morphogenesis of the tip epithelium in the multicellular stage of the *Dictyostelium* life cycle.

### 4.3. DGAP1 and GAPA Act Antagonistically in the Regulation of the Actin Cytoskeleton in Dictyostelium

It appears, therefore, that DGAP1 and GAPA function as a pair of antagonistic proteins that oppositely regulate actin polymerisation and the assembly of contractility kits in the cortical actin cytoskeleton in *Dictyostelium*. In both processes, DGAP1 acts as a suppressor of GAPA. Given their high structural similarity, it is currently unknown which structural features determine these stark functional differences. Although their interactomes have not been systematically compared, the known and suspected binding partners of the two proteins largely overlap. It has been proposed that GAPA may be regulated by Ca^2+^ because it purportedly contains a partly conserved IQ motif that could mediate its binding to calmodulin [38]. Although the binding of GAPA to calmodulin has not been experimentally tested, we consider it unlikely, as the hypothetical IQ motif would be atypically located within the GRD domain. DGAP1 and GAPA contain GRD, RGCt, and CT homology regions also found in yeast and metazoan IQGAPs, but they do not share their N-terminal actin-binding CH and coiled coil domains or multiple IQ repeats. Since cortexillins contain CH and coiled coil domains, it has been suggested that fungal and metazoan IQGAPs may correspond to fused versions of the complex between the truncated *Dictyostelium* IQGAPs, such as DGAP1 and GAPA, and cortexillins [51]. However, this hypothesis is not plausible because cortexillins harbour a CH1/CH2 tandem of the α-actinin/spectrin type, whereas IQGAPs contain a single CH3 domain [35,52,53,54]. On the other hand, it is possible that DGAP1 and GAPA arose from a full-length evolutionary predecessor with a domain organisation similar to mammalian IQGAPs through a domain loss mechanism. It remains to be determined whether these partial IQGAP orthologues from *Dictyostelium* resemble ancestors or descendants of the ancient IQGAP prototype.

## 5. IqgC—A GAP Among the IQGAPs

IqgC is a RasGAP protein originally classified as a member of the IQGAP family. However, IqgC is more closely related to fungal GAP1 family proteins, which have a deceptively similar domain organisation (RasGAP-RGCt-CT) [55]. IqgC interacts with Ras, Rab, and Rap GTPases, but it exhibits GAP activity only towards Ras. It localises to macropinocytic and phagocytic cups, reduces macropinosome size, and negatively regulates macroendocytosis by deactivating RasG [56,57]. IqgC supports cell–substratum adhesion by stabilising ventral adhesion foci [58]. Its RGCt domain is critical for recruitment to adhesion sites, while the RasGAP domain regulates the turnover of the adhesion foci. IqgC also positively influences directed migration by stabilising adhesion foci and coordinating Ras-mediated actin polymerisation. IqgC orthologues are conserved across six other dictyostelid species, highlighting their universal importance in amoeboid physiology [59]. Altogether, IqgC appears to play an important role in balancing feeding and migratory behaviours in *D. discoideum*. For more details about IqgC, we refer the reader to a recent review article [59].

## 6. IqgD—The Only *Dictyostelium* IQGAP That Binds F-Actin

### 6.1. IqgD—A Truncated IQGAP Fused to Fimbrin?

IqgD is the largest *D. discoideum* IQGAP because, in addition to C-terminal homology regions, it contains an N-terminal extension with CH domains. Hence, IqgD, like mammalian IQGAPs, binds Rac1 and F-actin [60]. Notably, IqgD harbours two fimbrin-type CHDs, CHf1 and CHf2, which together form an actin-binding domain (ABD) [35]. This domain is related to the first fimbrin ABD and distinguishes IqgD from mammalian IQGAPs, which bind F-actin via a single type 3 CHD [53]. This chimeric domain organisation, comprising a duplex CHD typical of the fimbrin family and a GRD-RGCt-CT triad typical of the IQGAP family, suggests that IqgD evolved through the addition of a fimbrin to a typically truncated *Dictyostelium* IQGAP [35]. Accordingly, the unique fimbrin-related proteins of *D. discoideum* are thought to have resulted from extensive genomic reorganisations, possibly involving retroposition events [61]. Retrotransposons make up about 8% of the *D. discoideum* genome [62], which is less than in most animals but relatively high compared to other dictyostelids [63]. Furthermore, during the generation of *iqgD^−^* cells, we serendipitously uncovered a genomic locus named *iqgD^L2^*, which encodes an IqgD paralogue. The partial sequence of *iqgD^L2^* is identical to the known *iqgD^L1^*, except that all three introns present in *iqgD^L1^* are absent in *iqgD^L2^*. Transcription from *iqgD^L2^* (*iqgD^L1−^* cells) was several hundred times lower than from *iqgD^L1^* and *iqgD^L2^* combined (wild-type cells) but still detectable [60]. We hypothesise that the *iqgD^L2^* locus corresponds to a gene that has arisen by retroposition—a retrogene. In addition to the typical lack of introns, it has been suggested that many retrogenes, particularly young ones, tend to have lower transcription levels [64]. Another diagnostic feature of putative retrogenes is their chimeric architecture, originating from the fusion of exons from separate genes, which we speculate had already occurred with the original gene at the *iqgD^L1^* locus.

### 6.2. IqgD Is Essential for Normal Growth on Bacterial Lawns

IqgD binds three highly homologous *D. discoideum* Rac1 GTPases (Rac1A, Rac1B, and Rac1C) in their active forms. Based on experiments with human Rac1, which shares more than 80% overall identity and has a 100% identical effector domain with *Dictyostelium* Rac1s, IqgD maintains Rac1 GTPases in the active state, similar to mammalian IQGAPs [60,65,66,67]. Bimolecular fluorescence complementation experiments in live cells showed that the CT domain is essential, and the GRD significantly contributes to GTPase binding. Interestingly, IqgD, like DGAP1 and GAPA, also interacts with cortexillins I and II, and these interactions are mediated by both the GRD and CT domains [60]. Consistent with this, IqgD bound to active Rac1A cannot simultaneously bind cortexillins. This suggests that Rac1 GTPases regulate IqgD by a mechanism different from that used to regulate DGAP1 and GAPA.

IqgD is localised across the entire cell cortex, where it co-localises with F-actin and is highly enriched in macropinocytic and phagocytic cups. Surprisingly, IqgD deficiency does not affect fluid uptake or phagocytosis of bacteria in suspension. However, IqgD-deficient cells show markedly reduced growth on bacterial lawns, as indicated by significantly smaller plaque diameters and reduced cell size. Moreover, IqgD-deficient cells phagocytose surface-attached microbeads less efficiently than wild-type cells. Additionally, mutant cells exhibit reduced cell–substratum adhesion. Interestingly, IqgD and active Rac1 localise to F-actin-rich rings that form around surface-attached particles on the ventral side of the cell in contact with the substratum. A similar structure was recently described on the ventral side of macrophages around surface-attached particles at the cell–substratum interface [68]. This phagocytic adhesion ring (PAR) was shown to depend on integrin tension and Arp2/3-mediated actin polymerisation, and its role in the detachment of surface-bound particles was demonstrated. Although the analogous ring structure identified in *Dictyostelium* awaits detailed characterisation, it is possible that IqgD plays a role in substratum-specific phagocytosis, which is important for both *Dictyostelium* feeding and the removal of pathogens from metazoan tissues. However, the full functional role of IqgD in efficient growth on bacterial lawns remains to be elucidated.

## 7. Summary and Outlook

### 7.1. Evolutionary Relationship Between Animal and Dictyostelium IQGAPs

Based on their structure and biochemical activity, DGAP1, GAPA, and IqgD are partial orthologues of animal IQGAPs in *Dictyostelium*, while IqgC is the sole representative of the fungal GAP1 family of RasGAPs in this organism. The crucial difference between IqgC and the other three proteins is that the “GRD” of IqgC is a fully functional RasGAP domain, whereas the typical GRDs of the other three proteins lack Ras binding and RasGAP activities, as in animal IQGAPs. It remains an open question whether these truncated IQGAPs from *Dictyostelium* evolved by domain loss or whether animal IQGAP proteins evolved by domain gain from a common ancestor. Phylogenetic analysis indicates that the common ancestor of fungi and animals (Opisthokonts) likely possessed a relatively simple RasGAP [26]. This ancestral protein likely consisted of the RasGAP-RGCt-CT triad and probably represents the common progenitor of all IQGAPs, as well as fungal and protozoan GAP1 RasGAPs. In this scenario, fungal and protozoan GAP1 proteins, such as IqgC, have largely maintained this ancestral, streamlined architecture. In contrast, mutations of crucial amino acid residues led to the conversion of the RasGAP domain into GRD, giving rise to a proto-IQGAP whose domain architecture is largely preserved in DGAP1 and GAPA. Further domain gain events resulted in the fusion of additional modules to the N-terminus of this ancestral IQGAP, with an apparent preference for actin-binding domains. In the progenitor of animal IQGAPs, a single type 3 CHD was appended, whereas a duplex CHD typical of fimbrins was appended in IqgD.

### 7.2. Functional Differences Between Animal and Dictyostelium IQGAPs

Regardless of the phylogenetic relationship between animal and *Dictyostelium* IQGAPs, their different architectures lead to distinct roles in cell signalling in these evolutionarily distant organisms. The N-terminal half of IQGAP1 interacts with receptor tyrosine kinases (RTKs) and G protein-coupled receptors (GPCRs), modulating their expression, activation, and trafficking [6]. Animal IQGAPs also regulate growth factor-dependent signalling modules by scaffolding signalling complexes at activated receptors. For example, several RTKs, Rafs, MEK1/2, and ERK1/2 bind to IQGAP1 at its IQ domain [69,70,71,72,73,74,75,76]. *Dictyostelium* lacks conventional RTKs upstream of MAPK signalling [33] and has no clear homologue of Raf [77]. The animal Hippo/YAP signalling pathway is partially regulated by GPCRs but is not present in *Dictyostelium* in its canonical form [78]. IQGAP1 binds to and modulates the activity of core proteins of this pathway, such as LATS1 and YAP, which are absent in *Dictyostelium*, via its IQ domain [79,80]. The observation that MST2, LATS1, and YAP bind to the same domains of IQGAP1 as the kinases of the Raf–MEK–ERK cascade suggests that this scaffold may be important for regulating crosstalk between these two signalling modules. Overall, it appears that the major regulatory pathways supported by the N-terminal half of animal IQGAPs are not conserved in *Dictyostelium*.

IQGAP1 facilitates the PI3K/Akt pathway by recruiting phosphoinositide 3-kinase (PI3K), which converts phosphatidylinositol-4,5-bisphosphate (PIP_2_) to phosphatidylinositol-3,4,5-trisphosphate (PIP_3_), as well as kinases operating upstream of PI3K that produce PIP_2_ from phosphatidylinositol [81]. IQGAP1 also interacts with PDK1 and Akt recruited to PIP_3_, facilitating PDK1-mediated phosphorylation and activation of Akt [81]. IQGAP1 associates with mTORC2, further facilitating Akt activation [82]. IQGAP1 also interacts with mTORC1 and acts as a TORC1-scaffolding protein that modulates the mTORC1-S6K1-Akt1 negative feedback loop [83]. These interactions are mediated by N-terminal regions of IQGAP1 [81,84]. Both PI3K/Akt and mTORC pathways are present in *Dictyostelium* and play important roles in growth, chemotaxis, and development, but IQGAP-related proteins are apparently not involved [85,86]. Therefore, some regulatory pathways supported by the N-terminal half of animal IQGAPs are rewired in *Dictyostelium*.

### 7.3. The Actin Cytoskeleton Is a Common Target of Animal and Dictyostelium IQGAPs

Although IQGAPs are clearly involved in regulating actin dynamics, the detailed molecular mechanisms underlying their activity remain partly unresolved. Animal IQGAPs bind directly to actin filaments through their N-terminal CHD. Early studies showed that IQGAPs can act as F-actin cross-linkers when they oligomerise, a process enhanced by their interaction with activated Cdc42 or Rac [87,88]. Although it was proposed that IQGAPs stimulate N-WASP/Arp2/3-mediated actin polymerisation [89,90], this hypothesis was later challenged [91]. The relationship between IQGAP1 and formin-mediated actin polymerisation is also controversial. One group proposed that IQGAP1 is a calmodulin-regulated capper of microfilament barbed ends [91]; others suggested that IQGAP1 enhances and spatially restricts the actin-nucleating activity of Diaphanous-related formin 1 [16,92], while a third model proposes that IQGAP1 acts as a displacement factor for formins, thereby transiently stalling elongation of the microfilament plus end [93]. Single-molecule imaging of IQGAP1 showed that it forms dimers that laterally associate with microfilaments [94]. In this way, IQGAP1 dimers can organise microfilaments into thin bundles and stabilise them against depolymerisation. IQGAP1 was also shown to transiently cap barbed ends to stall microfilament growth [94].

Much less is known about the mechanisms by which IQGAP-related proteins regulate the actin cytoskeleton in *Dictyostelium*. As GAPA and DGAP1 lack an actin-binding domain, they interact with microfilaments only indirectly through their binding partners, e.g., cortexillins, and this interaction is crucial for efficient cytokinesis in *Dictyostelium* [95]. Similarly, the interaction of IQGAP3 with anillin is required for the localisation of IQGAP3 to the cleavage furrow and for the completion of cytokinesis in HeLa cells [96]. IqgD binds directly to microfilaments via its CHD, but it is not known whether or how this influences actin dynamics. Future studies should address whether IqgD oligomerises and whether it binds to the sides or barbed ends of microfilaments. Nevertheless, as IqgD knockout does not affect the quantity of F-actin in the cells, it is unlikely that it regulates microfilament polymerisation from the barbed end [60]. Although IQGAPs lack GAP activity towards Rho GTPases, they can influence the ratio between their active and inactive forms. It has been found that IQGAP1 significantly increases the levels of active, GTP-bound Cdc42 in the cell, resulting in the formation of peripheral actin microspikes [97]. In *Dictyostelium*, IqgD was shown to maintain Rac1 GTPases in the active state, whereas DGAP1 does not affect the intrinsic GTPase activity of Rac1 GTPases [41,60]. No comparable data exist for GAPA.

### 7.4. Open Questions and Future Directions

We must note that the presented 3D structures of *Dictyostelium* proteins are computational predictions that require experimental validation. Although proteins with more than 800 residues, such as GAPA and DGAP1, can probably be crystallised, this is challenging because several flexible domains may prevent the orderly packing required for crystal formation. Therefore, the use of the Cryo-EM approach is likely the recommended route, as the structures of proteins of approximately 90 kDa can be solved at near-atomic resolution [98]. We hypothesise that hybridisation between the predicted HN and RGCt-HC α-helices could contribute to the stability of a compact tertiary structure in the examined proteins. Its disruption may, therefore, play a role in regulating their activity; for instance, in the binding of Rho GTPases by the recently proposed two-step mechanism [10,12]. This hypothesis can be tested by assessing their interaction in vitro with isolated wild-type and heptad repeat-directed mutant polypeptides or by structural and functional studies using deletion mutants lacking HN or RGCt-HC α-helices.

It was shown for IQGAP1 that the polypeptide consisting of amino acid residues 956–1274, which closely corresponds to GRD, interacts with the polypeptide 1276–1657, which encompasses RGCt and CT [36]. Phosphorylation at Ser1443, located within the RGCt-H3 subdomain, strongly reduced this interaction. It was hypothesised that in vivo phosphorylation of IQGAP1 causes opening of the structure of the IQGAP1 C-terminus. The part of the sequence covering the putative HN helix was not included in this interaction study. The authors conclude that the tertiary structure of the IQGAP1 C-terminal half probably determines the differential binding properties of IQGAP1 with Cdc42 in its GTP-bound, GDP-bound, and nucleotide-depleted forms [36]. It was further shown that the region consisting of the 522 N-terminal amino acid residues of IQGAP1, which encompasses the CC domain, interacts with the C-terminal polypeptide 956–1657 [89]. Taken together, these findings suggest that in quiescent mammalian cells, full-length IQGAP1 adopts an inactive conformation, in which the two parts of the C-terminal half interact with each other and further interact with the N-terminal half of the protein [99]. It would be worthwhile to test whether comparable interactions also occur in *Dictyostelium* IQGAPs and whether the putative HN and RGCt-HC participate. It is quite possible that the 3D structure predicted by AlphaFold corresponds to the closed, autoinhibited conformation of *Dictyostelium* proteins.

Conservation of regulatory motifs, such as the polybasic stretch of interspersed lysine residues between the RGCt-HC and CT domains and the exposed serine residues in the RGCt-H3 domain, suggests that binding to PIP_2_ and serine phosphorylation are conserved in *Dictyostelium* IQGAPs. The possible functional consequences of these regulatory mechanisms need to be experimentally assessed, but they probably resemble similar mechanisms described in mammalian IQGAPs [17,36]. Except for IqgC, interactions with phospholipids have not been examined. Systematic analyses of protein–phospholipid interactions should, therefore, be performed using lipid-binding assays with full-length proteins and variants carrying mutations in lysine residues that constitute the putative polybasic region. Similarly, phosphorylation studies could clarify the role of phosphorylation in regulating the activity of *Dictyostelium* IQGAP-related proteins. The use of phosphomimetic and non-phosphorylatable mutants would help determine the proposed functional significance of the serine residues within the RGCt-H3 subdomain. Another possible avenue to investigate this question is to perform computational experiments by systematically exchanging serine residues for either phosphomimetic or neutral surrogates and studying the consequences on the predicted structure. However, it is known that AlphaFold struggles to predict open protein conformations, particularly when the closed state is thermodynamically favoured or more heavily represented in the training data [21].

The mechanistic basis for the functional antagonism between the highly similar proteins DGAP1 and GAPA remains unclear. For example, why do these two proteins influence actin polymerisation and the assembly of contractile complexes in opposite ways? Interestingly, IQGAP1 and IQGAP2 also provide an example of two similar proteins with opposing cellular functions. Whereas IQGAP1 is implicated as an oncoprotein in many human cancers, IQGAP2 functions as a tumour suppressor [7,100]. Two mechanisms have been proposed for how IQGAP2 could block the binding of IQGAP1 to downstream effector molecules [99]. One possible mechanism is the competitive binding of IQGAP2 to common interactors, which would attenuate the cellular activities of IQGAP1. Another possibility is that IQGAP2 forms a complex with IQGAP1 and thereby blocks IQGAP1 interactions with its effectors. For example, as in the intramolecular interactions that lead to an autoinhibited conformation of IQGAP1, the two parts of the C-terminal half of the two proteins could interact with each other. These hypothetical mechanisms could also be responsible for the inhibition of GAPA by DGAP1.

Cell cycle-dependent phosphorylation of the fission yeast IQGAP, Rng2, has been shown to be involved in the assembly and stability of the contractile actomyosin ring during cytokinesis [101]. The transient phosphorylation of Rng2 appears to be important for contractile ring assembly in collaboration with myosin-II [102]. In another fungus, *Aspergillus nidulans*, the IQGAP orthologue SepG is required for constriction of the contractile ring [103]. Notably, these authors characterised a specific septation mutant resulting from a glycine-to-arginine amino acid substitution at position 1637, located in the predicted flexible IDR linker between RGCt-HC and CT. The strong effect of this single residue substitution in this region supports the importance of tertiary structure flexibility for the functionality of IQGAPs. Building on this concept, the novel structural insights obtained here through computational modeling with AlphaFold 3 could inform swapping experiments in which individual domains and linking regions are exchanged between GAPA and DGAP1, and the resulting functional consequences are assessed. This approach would also provide guidelines for further investigation of the interaction mechanisms of these truncated IQGAPs with cytoskeletal complexes, such as the proposed contractility kits. Moreover, it could assist in characterising the general roles of the domains in the C-terminal half of animal IQGAPs.

Finally, information about the role of IQGAP-related proteins, primarily IqgC and IqgD, in the multicellular stage of the *Dictyostelium* life cycle is scarce and requires further investigation. Developmental expression profiles indicate that IqgD is upregulated in late development. While all four proteins are specifically localised to prestalk cells in slugs, IqgD is predominantly expressed in terminally differentiated stalk cells, whereas IqgC is predominantly expressed in spore cells [104]. Additionally, IqgD is upregulated and IqgC is downregulated during encystation [104]. These distinct expression patterns suggest that IqgC and IqgD may play specialised, non-overlapping roles in coordinating cell fate and tissue morphogenesis during the final stages of development.

## Figures and Tables

**Table 1:** Amino acid stretches corresponding to domains predicted by AlphaFold 3 and shown in colour in Figure 1: N-terminal helix (HN), GAP-related domain (GRD), triple helix in the RasGAP C-terminal (RGCt) domain (RGCt-H3), C-terminal helix in the RGCt domain (RGCt-HC), and C-terminus (CT).

	HN	GRD	RGCt-H3	RGCt-HC	CT
DGAP1	110–157	180–544	576–641	652–715	730–822
GAPA	144–181	215–577	610–683	695–754	766–860
IqgC	53–98	126–487	552–613	626–687	725–817
IqgD	593–632	661–1016	1136–1196	1215–1279	1294–1385
IQGAP1	880–922	963–1331	1377–1468	1479–1542	1561–1657

## Data Availability

All new data created and analyzed in this study are included in the manuscript and Supplementary Materials.

## Supplementary Materials

The following supporting information can be downloaded at: https://www.mdpi.com/article/10.3390/ijms27125462/s1.

## Abbreviations

The following abbreviations are used in this manuscript:

ABD	Actin-binding domain
aPI	Atypical phosphoinositide-binding domain
CC	Coiled coil repeat region
CHD	Calponin homology domain
CT	Extreme C-terminal domain
GPCRs	G protein-coupled receptors
GRD	GTPase-activating protein (GAP)-related domain
HN	N-terminal helix
IDR	Intrinsically disordered region
IQ	Isoleucine- and glutamine-rich motif
LECA	Last eukaryotic common ancestor
PAE	Predicted align error (in AlphaFold)
PAR	Phagocytic adhesion ring
PI3K	Phosphoinositide 3-kinase
PIP_2_	Phosphatidylinositol-4,5-bisphosphate
PIP_3_	Phosphatidylinositol-3,4,5-trisphosphate
pLDDT	Predicted local distance difference test (in AlphaFold)
RGCt	RasGAP C-terminal domain
RGCt-H3	Triplet of short helices within the RGCt domain
RGCt-HC	C-terminal helix within the RGCt domain
RTKs	Receptor tyrosine kinases
WW	Tryptophan-containing proline-rich motif-binding region
