# Supplementary material for: Queue Gaps Among the IQGAPs in Dictyostelium discoideum
Source: Int J Mol Sci. 2026 Jun 17;27(12):5462. doi: 10.3390/ijms27125462 (PMC13299636; doi:10.3390/ijms27125462)
Supplement: Supplementary file 1 [file ijms-27-05462-s001.zip › ijms-4291603-supplementary.pdf]

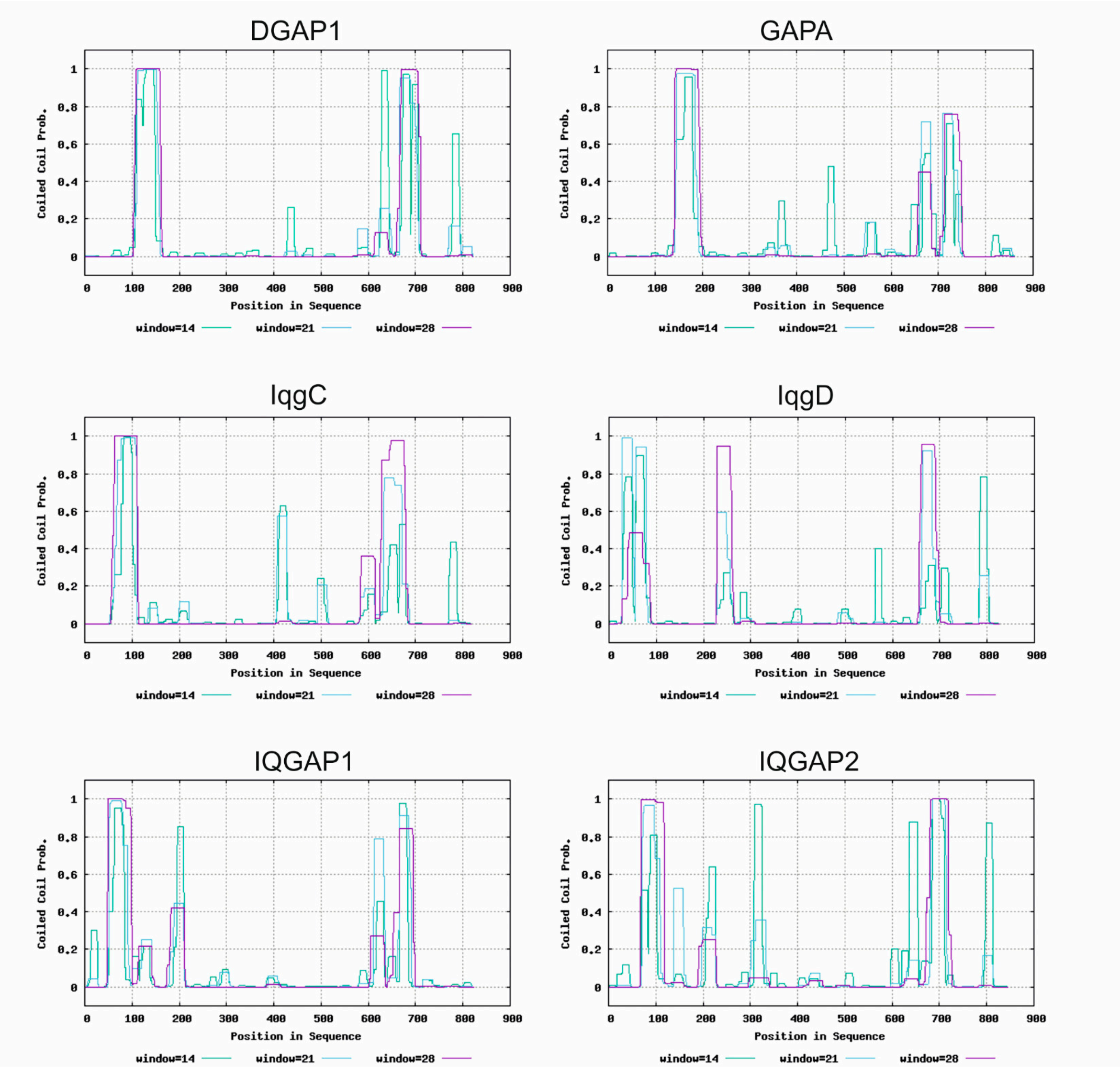

Figure S1. Probability of coiled coil structure formation as determined by the PCOILS algorithm using default parameters [20]. Calculated probabilities are shown for full-length DGAP1, GAPA, and IqgC; for segments of IqgD (560–1385) and IQGAP1 (835–1657) displayed in Figures 1B-C; and for a segment of IQGAP2 of comparable length (731–1575). For each protein, the pair of magenta peaks with the highest probability of coiled coil formation corresponds to the HN and RGCt-HC α-helices.

Table S1. The average predicted Local Distance Difference Test (pLDDT) values for amino acid stretches corresponding to the N-terminal helix (HN), GAP-related domain (GRD), triple helix in the RGCt domain (RGCt-H3), C-terminal helix in the RGCt domain (RGCt-HC), and C-terminal domain (CT), as presented in Figure 1 and Table 1. Also included are the average pLDDT values for two linker regions, which we hypothesise may represent Intrinsically Disordered Regions (IDRs), connecting the HN and GRD domain (IDR1) and the RGCt-HC and CT domains (IDR2).

	**HN**	**IDR1**	**GRD**	**RGCt-H3**	**RGCt-HC**	**IDR2**	**CT**
DGAP1	69	42	87	82	78	55	85
GAPA	74	46	88	78	80	57	82
IqgC	76	51	89	73	74	43	87
IqgD	74	52	78	75	77	54	85
IQGAP1	72	36	84	81	77	49	85
